# Noninvasive urinary protein signatures combined clinical information associated with microvascular invasion risk in HCC patients

**DOI:** 10.1186/s12916-023-03137-6

**Published:** 2023-12-05

**Authors:** Yaru Wang, Bo Meng, Xijun Wang, Anke Wu, Xiaoyu Li, Xiaohong Qian, Jianxiong Wu, Wantao Ying, Ting Xiao, Weiqi Rong

**Affiliations:** 1 Department of Hepatobiliary Surgery, National Cancer Center/National Clinical Research Center for Cancer/Cancer Hospital, Chinese Academy of Medical Sciences and Peking Union Medical College, Beijing, 100021 China; 2 State Key Laboratory of Molecular Oncology, Department of Etiology and Carcinogenesis, National Cancer Center/National Clinical Research Center for Cancer/Cancer Hospital, Chinese Academy of Medical Sciences and Peking Union Medical College, Beijing, 100021 China; 3 State Key Laboratory of Proteomics, National Center for Protein Sciences (Beijing), Beijing Proteome Research Center, Beijing Institute of Lifeomics, Beijing, 102206 China; 4 College of Life Science and Bioengineering, Beijing University of Technology, Beijing, 100124 China; 5 Center for Advanced Measurement Science, National Institute of Metrology, Beijing, 100029 China; 6 Department of Clinical Trial Research Center, Beijing Hospital, National Center of Gerontology, Institute of Geriatric Medicine, Chinese Academy of Medical Sciences, Beijing, 100005 China

**Keywords:** Hepatocellular carcinoma (HCC), Microvascular invasion (MVI), Urine proteomics, Biomarker diagnosis

## Abstract

**Background:**

Microvascular invasion (MVI) is the main factor affecting the prognosis of patients with hepatocellular carcinoma (HCC). The aim of this study was to identify accurate diagnostic biomarkers from urinary protein signatures for preoperative prediction.

**Methods:**

We conducted label-free quantitative proteomic studies on urine samples of 91 HCC patients and 22 healthy controls. We identified candidate biomarkers capable of predicting MVI status and combined them with patient clinical information to perform a preoperative nomogram for predicting MVI status in the training cohort. Then, the nomogram was validated in the testing cohort (*n* = 23). Expression levels of biomarkers were further confirmed by enzyme-linked immunosorbent assay (ELISA) in an independent validation HCC cohort (*n* = 57).

**Results:**

Urinary proteomic features of healthy controls are mainly characterized by active metabolic processes. Cell adhesion and cell proliferation-related pathways were highly defined in the HCC group, such as extracellular matrix organization, cell–cell adhesion, and cell–cell junction organization, which confirms the malignant phenotype of HCC patients. Based on the expression levels of four proteins: *CETP*, *HGFL*, *L1CAM*, and *LAIR2*, combined with tumor diameter, serum AFP, and GGT concentrations to establish a preoperative MVI status prediction model for HCC patients. The nomogram achieved good concordance indexes of 0.809 and 0.783 in predicting MVI in the training and testing cohorts.

**Conclusions:**

The four-protein-related nomogram in urine samples is a promising preoperative prediction model for the MVI status of HCC patients. Using the model, the risk for an individual patient to harbor MVI can be determined.

**Supplementary Information:**

The online version contains supplementary material available at 10.1186/s12916-023-03137-6.

## Background

HCC is one of the most common primary hepatic malignant tumors and its incidence is increasing worldwide [1]. It is the second leading cause of cancer-specific mortality in the Asia–Pacific regions, especially in China [2]. Surgical resection is the main way to treat HCC, but the high postoperative recurrence and metastasis pose a major challenge for the cure of HCC treatment [3]. Microvascular invasion (MVI) is one of the most important prognostic factors for HCC after surgical treatment, HCC patients with MVI have a higher potential for cancer metastasis [4]. Currently, the diagnosis of MVI is determined on histologic examination of the surgical specimens obtained after liver resection or transplantation [5]. Therefore, the influence of the diagnosis on preoperative decision-making is limited. Clinicians hope to choose surgical treatment with larger margins for HCC patients with MVI to prevent recurrence and metastasis of patients. An accurate preoperative estimation of MVI presence can help surgeons choose appropriate surgical procedures for patients based on risk–benefit assessment.

Various preoperative assessment models for MVI have been reported. For example, patient imaging characteristics are closely related to MVI [6]. However, these results require further validation to avoid interobserver variability. The genes that have been confirmed to be associated with tumor vascular invasion are less applicable in the preoperative risk assessment of MVI [7]. Studies have also proposed the use of serum or tumor biomarkers to estimate MVI risk. However, these serum markers may also be elevated in patients with various benign liver diseases, and serum markers lack specificity [8].

Urine is a non-invasive and easily obtained body fluid, which is desirable to be used as a new way to find biomarkers. Some urine proteins have been used as biomarkers for the diagnosis of certain diseases [9]. For example, urinary *NMP22* (nuclear matrix protein–22) has already been approved by FDA for diagnosis of bladder cancer [10]. Urinary proteomics also has a broad role in the discovery of biomarkers of lung cancer, heart failure, and urogenital diseases [11, 12]. Recent studies have also confirmed that urinary protein could be used as biomarkers for the diagnosis of HCC [13], but until now, there is still no available urinary biomarker of HCC with MVI. The discovery of HCC urinary protein markers for pre-operation prediction of MVI is still needed.

Here, we report the discovery of a promising prognostic index for HCC MVI. First, we used label-free quantitative proteomics to assess HCC-associated proteomic changes. Second, we selected the candidate biomarkers in the training cohort of HCC patients that could distinguish patients’ MVI status using LASSO-logistic regression. At the same time, we combined clinical information to establish a nomogram that could predict the patients’ MVI status. Third, we verified the distinguishing performance of the nomogram in the testing cohort in HCC patients. Finally, the expression levels of candidate biomarkers were further validated in an independent validation cohort using ELISA. This stepwise study yields highly accurate noninvasive urinary protein signatures and will improve the application of urinary proteomics in future HCC research.

## Methods

### Study design

**Fig. 1:**
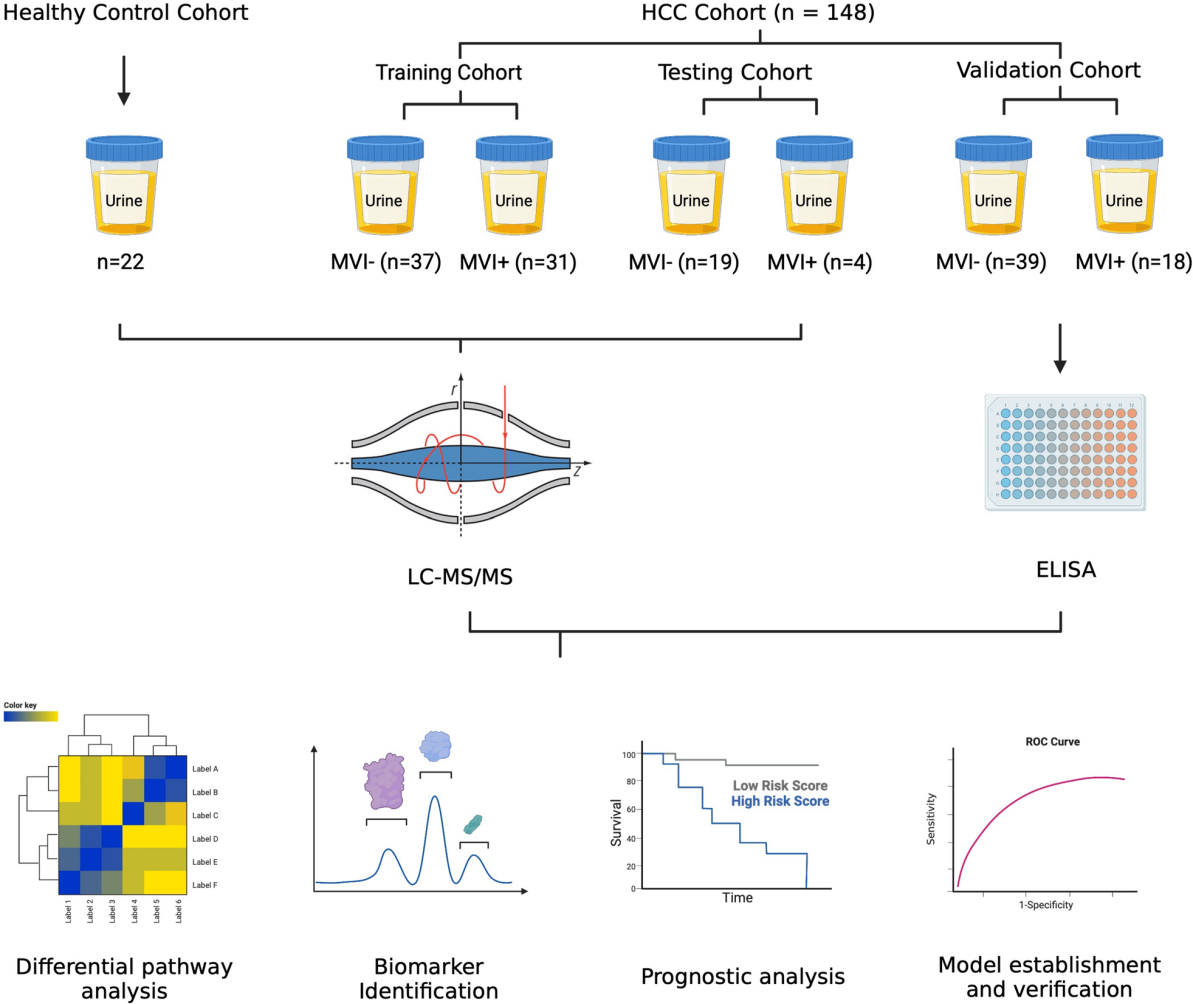
Study design. First, the urine landscape between HCC patients and healthy controls was described using proteomics. Second, a nomogram for predicting the MVI status of HCC patients before surgery based on the urinary proteome was constructed. Third, ELISA was used to verify the accuracy of the model

The objectives of the present study were to systemically identify and validate potential noninvasive MVI predictive markers for HCC in urine. As shown in Fig. 1, label-free quantitative proteomics analyses were performed for 91 HCC patients (35 MVI positive and 56 MVI negative) and 22 healthy controls. Then, 57 HCC patients of the validation cohort were enrolled for further verification of screened biomarkers. This study was approved by the Ethics Committee of the Institute of Basic Medical Sciences, Chinese Academy of Medical Sciences with an exemption of informed consent, and was performed according to the Declaration of Helsinki Principles.

### Patients and healthy controls

A total of 148 HCC patients were recruited from the Cancer Hospital, Chinese Academy of Medical Sciences, from 2018 to 2021. All patients were pathologically diagnosed by two senior pathologists, and random morning midstream urine samples were collected prior to surgical operations. The patients were divided into the training cohort, test cohort, and validation cohort. The training cohort contains 31 MVI-positive HCC patients and 37 MVI-negative HCC patients; the test cohort contains 4 MVI-positive HCC patients and 19 MVI-negative HCC patients; the validation cohort contains 18 cases of MVI-positive HCC patients and 39 cases of MVI-negative HCC patients. Both the training cohort and the test cohort were subjected to LC–MS/MS, and the validation cohort was subjected to ELISA experimental verification. The detailed clinical characteristics of these patients are shown in Additional file 1: Table S1. In brief, the mean ages of training and testing cohort patients were 59.43 and 61.26 years, 86.8% and 82.6% were males (Additional file 1: Table S1).

In addition, 22 urine samples from Healthy controls were obtained from the Health Medical Center of the Cancer Hospital. The enrollment criteria for Healthy Control subjects were as follows: (1) the absence of benign or malignant tumors, (2) a qualified physical examination finding no dysfunction of vital organs, and (3) normal renal function and without albuminuria. After collection, urine samples were stored at − 80 °C.

### Determination of MVI

The definition of MVI status depends on the pathological diagnosis of patients with HCC after R0 resection. According to the characteristics of MVI of HCC, “Guidelines for Standardized Pathological Diagnosis of Primary Liver Cancer (2015 Edition)” defines MVI as cancer cell nests seen only in the vascular lumen lined with endothelial cells under the microscope, mainly with branches of the portal vein. In the guideline, MVI is divided into two types, M1 and M2. M1 is defined as less than or equal to 5 cancer nests, and it is more likely to occur in the adjacent (< 1 cm) liver tissue area. M2 means that more than 5 cancer nests can be seen under the microscope, or the cancer nests occur in the distant liver tissue area (greater than 1 cm). The MVI-negative and positive group definitions mentioned in our study strictly followed this rule and were interpreted by two senior pathologists. In this study, M1 and M2 were combined for analysis, and we have added detailed information in the Additional materials.

### Sample preparation

Midstream urine was collected from either healthy individual or HCC patients, and centrifuged at 12,000 g, 4 ℃ for 30 min. The supernatant was recovered and mixed with acetone with a volume ratio of 1:4 and put at − 20 ℃ overnight. The proteins were recovered by centrifugation at 5000 g for 5 min and resolved in UA buffer (8 M urea, 100 mM Tris–HCl, pH 8.5). Quantitation of protein concentration was performed by the Bradford approach. Trypsin digestion was performed using filter-aided sample preparation (FASP).

### DDA library building and DIA acquisition

Urine peptides from 6 healthy subjects and 6 HCC patients were mixed respectively and used for building the peptide library using DDA analysis. DDA library building and following DIA analysis were performed with thermo Q Exactive HF coupled to an EASY-nano-LC 1000 system. The system includes a 100 μm I.D. × 30 cm column (C18, 1.9 μm, 120 Å, Dr. Maisch GmbH). The mobile phase consisted of 0.1% formic acid in water (A) and 0.1% formic acid/100% acetonitrile (B). For the DDA library building, the mobile phase flow rate was 500 nl/min and subjected into a gradient profile which was set as follows: 6–10% buffer B for 13 min, 10–23% buffer B for 86 min, 23–33% buffer B for 21 min, 33–90% buffer B for 3 min, 90% buffer B for 12 min. The Q Exactive HF mass spectrometer parameters were as follows: MS spectra were collected from 375 to 1400 m/z at a resolution of 120 K along with data-dependent Orbitrap HCD MS/MS spectra at a resolution of 15 K. Ions selected for MS/MS were fragmented using a normalized collision energy of 27%. Dynamic exclusion time was set to 18 s. For DIA analysis, the mobile phase and durations were as follows: 4–8% buffer B for 13 min, 8–22% buffer B for 86 min, 22–31% buffer B for 21 min, 31–90% buffer B for 3 min, 90% buffer B for 12 min; the flow rate was 600 nl/min. Peptides were analyzed with one full scan (400–1200 m/z) with 60,000 resolution and AGC target of 3e6. For DIA acquisition window and mass spectrometry parameters, 400–800 m/z was divided into 20 windows, each window was 20 Da (AGC target of 3 × e6, maximum IT 41 ms, loop count 20, NCE 27%); 800–1000 m/z was divided into 5 windows, each window was 40 Da (AGC target of 3 × e6, maximum IT 41 ms, loop count 5, NCE 27%); 1000–1200 m/z was divided into 5 windows, each window was 50 Da (AGC target of 3 × e6, maximum IT 41 ms, loop count 4, NCE 27%).

### Data analysis

The mass spectrometry files used to build the library were analyzed by MaxQuant (v1.6.2.19), While the DIA MS data were processed using DIA-NN (1.7.6) in the library-dependent mode. Both software uses the human database downloaded from uniprot (SwissProt September 11, 2020, 20375 entries). For MaxQuant, the parameters used included trypsin as a digestive enzyme, two missing cleavage sites, and cysteine carboxyaminomethylation as a fixed modification. DIA-NN was used with recommended settings (each peptide allows one missing cleavage and a maximum of two variable modifications, acetylation of protein N-termini or/and oxidation of methionine).

### LC–MS/MS data processing

Prior to LC–MS/MS analysis, we imputed and normalized the data. To ensure the accuracy of imputation, we first removed proteins with missing expression values in more than 50% of participants. Subsequently, for the remaining proteins with missing values, we imputed them using their minimum value.

### Identification of representative modules of urine samples with different characteristics

Weight gene co-expression network analysis (WGCNA) was applied to identify the biological changes in the MVI-positive HCC patients, MVI-negative HCC patients, and Healthy controls. All proteins were evaluated to construct the coexpression network. The “WGCNA” R package and R tutorials were used to construct the weighted protein coexpression network. The R package “clusterProfiler” was applied for the KEGG analysis of four core transcriptional modules inferred from the WGCNA.

### Nomogram building

According to the ratio of 3:1, HCC patients were divided into a training cohort (*n* = 68) and a testing cohort (*n* = 23) by random sampling. To further select the proteins related to MVI status with HCC patients, we used the Lasso-Logistic regression model to select the most useful diagnostic markers of all the proteins identified with the training cohort and constructed a four-protein-based score.

Based on the Logistic regression model of protein expression level, if the coefficient *β* of each candidate protein were obtained, then each patient would get a protein score as follows:

$$\text{Protein Score}=\sum\limits_{i=1}^{4}\left({G}_{i}*{\upbeta }_{i}\right)$$

G_i_ was the normalized expression value of protein_i_, and β_i_ was the regression coefficient of protein_i_ in the multivariate logistic regression analysis. The Youden index of protein score as cutoff value classified patients with HCC into high-risk and low-risk MVI groups (cutoff value = 21.53).

Univariate logistic regression was performed to screen the preoperative clinical information of HCC patients that can enter the model construction (*p* < 0.05), and the screened clinical information and protein score were combined to construct a multivariate logistic regression model and establish the nomogram which could predict the MVI status of HCC patients.

### ELISA

Urine *CETP*, *HGFL*, *L1CAM*, *LAIR2*, and creatinine concentrations were assessed by ELISA kits (*CETP*, *L1CAM*, *LAIR2*, creatinine: USCN, China. *HGFL*: abcam, USA) according to the manufacturer’s instructions. Briefly, for *CETP*, *L1CAM*, *LAIR2*, and creatinine, 100 µL urine was added into wells on a microplate and incubated at 37 °C for 2 h. Then, 100 µL biotinylated detector antibody was added to each well and incubated at 37 °C for 1 h. After 3 washes, 100uL conjugate was added to each well and incubated at 37 °C for 30 min. The 100 µL stop solution was added to each well and immediately read OD at 450 nm (Bio-Rad Laboratory, Hercules, CA, USA). For *HGFL*, 50 µL urine and 50 µL antibody cocktail were added into wells on a microplate, then incubated at 37 °C for 1 h. After 3 washes, 100 µL conjugate was added to each well and incubated at 37 °C for 30 min. The 100 µL stop solution was added to each well and immediately read OD at 450 nm (Bio-Rad Laboratory, Hercules, CA, USA).

Urine concentrations of *CETP*, *HGFL*, *L1CAM*, and *LAIR2* were calibrated by the corresponding urine creatinine measurement.

### Statistics

All statistical analyses were performed using R (https://www.r-project.org). The Wilcoxon rank-sum test was adopted to compare differences between the two groups. The receiver operating characteristic (ROC) analysis was performed to investigate the predictive accuracy of each feature and the nomogram using the R package “ROCR”. Survival probabilities were estimated with the Kaplan–Meier method, and the log-rank test was used to compare the survival distributions between the two groups. A log-rank *P* < 0.05 was considered statistically significant.

## Results

### Clinical characteristics of urine specimens

A total of 170 subjects, including 22 healthy controls and 148 HCC patients with/without MVI, were recruited for this study. Urine samples from HCC patients were collected before surgery. The detailed clinical information of representative tissues of HCC patients undergoing urine proteomics is shown in Additional file 1: Table S1. The MVI status of all HCC patients was assessed by two pathologists. We stained the postoperative pathological sections of the patients with hematoxylin and eosin (H&E) staining. In the pathological sections of the patients in the MVI-positive group, cancer nests could be seen in the vascular lumen (Additional file 1: Fig. S1A, Additional file 2). We performed a survival analysis to investigate the relationship between MVI status and prognosis of patients with HCC. Relevant prognostic information was available for patients with HCC enrolled in this study, including recurrence, metastasis, and survival status. The Kaplan–Meier analysis showed significant differences in prognosis between patients with MVI negative and positive HCC. A longer disease-free survival (DFS) time was observed in patients with MVI-negative HCC than in patients with MVI-positive HCC (Additional file 1: Fig. S1B). While the overall survival (OS) time of MVI-negative patients was also longer, the difference was not statistically significant due to the small sample size or postoperative treatment received by patients (Additional file 1: Fig. S1C).

### Urinary proteomics analysis

Urinary protein candidates were discovered from the urinary proteins of 91 primary HCC patients and 22 healthy controls by label-free quantitative proteomics using mass spectrometry-based sequencing with the data-independent acquisition (DIA) mode. 91 HCC patients were divided into training (68 cases, 75%) and testing cohorts (23 cases, 25%) using randomization.

The present study collected a total of 91 primary HCC patients’ urine samples before the surgery and 22 healthy controls’ urine samples as the control. A schematic of the experiment is shown in Fig. 1. During the discovery age, 2279 proteins were identified in label-free proteomics experiments. There were no significant differences in the number and expression abundance of proteins identified in the urine of HCC patients and Healthy controls (Additional file 1: Fig. S2B). Partial least squares discriminant analysis (PLS-DA) was performed to distinguish the HCC patients and Healthy controls, results showed that there was a significant difference in urine samples between healthy controls and HCC patients, and the MVI-negative group could still cluster together in the HCC patients (Additional file 1: Fig. S3A).

### Urinary proteomic signatures of HCC and healthy controls

**Fig. 2:**
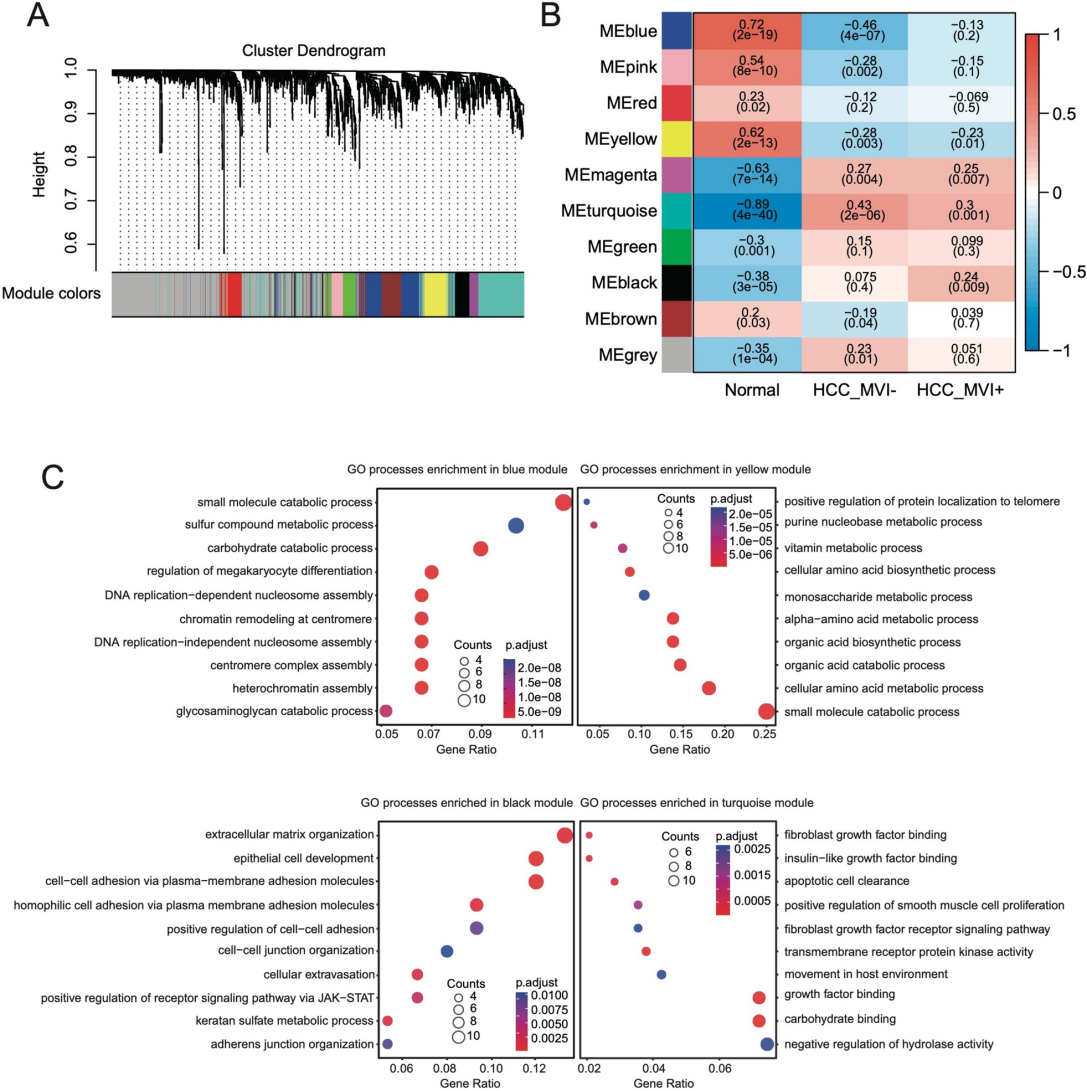
Identification of modules representative of HCC and healthy controls. **A** Hierarchical cluster dendrogram of the proteins with the highest standard deviation. The identified modules underneath the tree are color coded. **B** Heatmap of module-trait associations; rows represent the module eigengene, and columns represent clinical traits. The Spearman correlation and significance level enclosed in brackets are labeled in each cell. The color intensity of the cell corresponds to the correlation coefficient. **C** Dot plot of the biological process enrichment results. The dot size and color represent the gene count and enrichment level, respectively

To systematically characterize the expression patterns of urinary proteomics of HCC and Healthy controls, WGCNA was performed on the urine proteomic and identified 10 protein modules with all proteins (Fig. 2A, Additional file 1: Fig. S3B). In relating these modules to group information by correlating the eigengenes of each module with compartment traits. Four modules with the most significant correlations to the HCC-MVI negative (module turquoise), HCC-MVI positive (module black), and healthy controls (module blue and module yellow) were identified (Fig. 2B). The heatmap in the figure shows the expression levels of proteins in the above several modules, and these four modules have remarkably high expression levels with their most correlated samples. Given the representativeness of these four modules, biological process enrichment analysis was applied to investigate the related properties in the process of tumorigenesis (Fig. 2C). The blue module and yellow module, which had remarkably high expression levels in the Healthy controls’ urine samples, were characterized by the overexpression of proteins involved in the catabolic process, DNA replication, and megakaryocyte differentiation process. Cell adhesion-related pathways such as extracellular matrix organization, cell–cell adhesion, and cell–cell junction organization were highly expressed in the black module, whose expression level was the highest in the HCC-MVI positive group. Several biological processes related to cell proliferation such as growth factors binding and smooth muscle proliferation were enriched in the turquoise module. The systematic analysis of HCC patients with different MVI statuses and healthy controls indicated that the metabolic process was active in the urine samples of Healthy controls, while the cell adhesion process was relatively high in the urine samples of HCC patients with MVI, and the cell proliferation process was active in the urine samples of HCC patients with negative MVI.

### Selection of candidate biomarkers

**Fig. 3:**
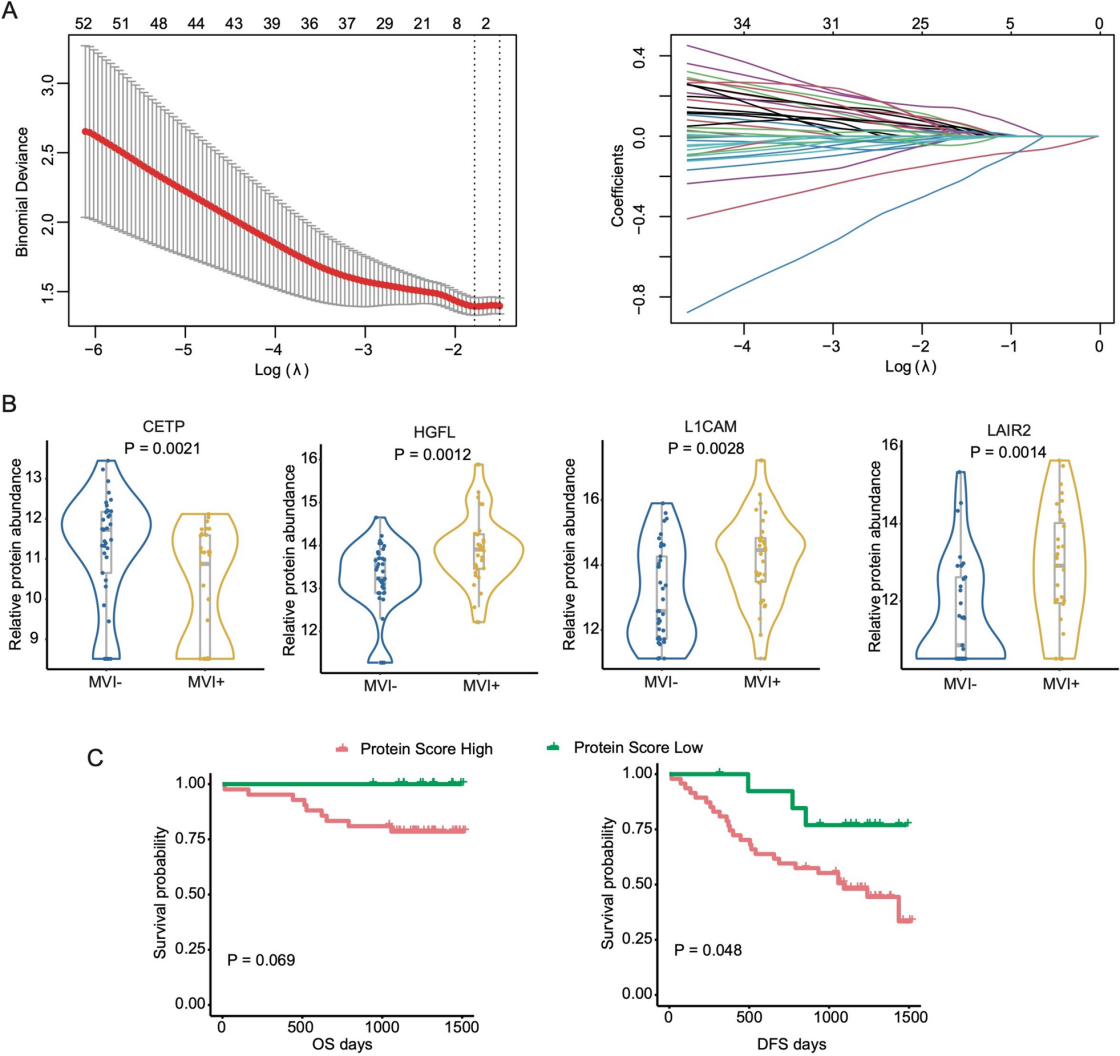
Construction of a model by urinary protein characteristics of HCC patients with different MVI statuses. **A** LASSO coefficient profiles of urine proteins. **B** The expression level of candidate biomarkers in different MVI status HCC patients. **C** Survival analyses of HCC patients with high or low protein score. Left panel, OS; right panel, DFS

The workflow for constructing the prediction of MVI status using urine protein signatures is as follows. First, 1791 proteins were input into LASSO-Logistic regression analysis in the training dataset (Fig. 3A), and 4 biomarkers with non-zero coefficients were obtained. Subsequently, pairwise correlation analysis was performed on these proteins, and we found that none of the proteins had similar expression patterns (Additional file 1: Fig. S4A). Therefore, we used the *CETP*, *HGFL*, *L1CAM*, and *LAIR2* for further model construction (Additional file 1: Fig. S3D, Additional file 1: Fig. S4B, Additional file 3). The biological functions and their roles in cancer progression were also reported in previous studies. *CETP* is involved in lipid transport, and its abnormal expression suggests that there may be metabolic reprogramming in different MVI states, which deserves further study [14]. *HGFL* is a hepatocyte growth factor-like protein, and its high expression in the MVI-positive group is consistent with the stronger proliferative ability in HCC tissues of MVI-positive patients [15]. As one of the neural adhesion molecules, *L1CAM* is expressed in many human cancers and is often associated with poor prognosis [16]. *LAIR2* is a secreted protein that promotes tumor immune evasion by interacting with collagen, and some studies have focused on its application as a tumor immunotherapy target [17]. The expression levels of these proteins were analyzed between MVI negative and positive groups (Fig. 3B). The Youden index of protein score as the cutoff value classified patients with HCC into high-risk and low-risk MVI groups. Likewise, we investigated the protein score in predicting patients’ survival, a high protein score can indicate a poor prognosis of patients with HCC (Fig. 3C). At the same time, a single protein can also indicate the prognosis of patients to a certain extent (Additional file 1: Fig. S4C).

### Development and validation of the preoperative MVI-prediction nomogram

**Fig. 4:**
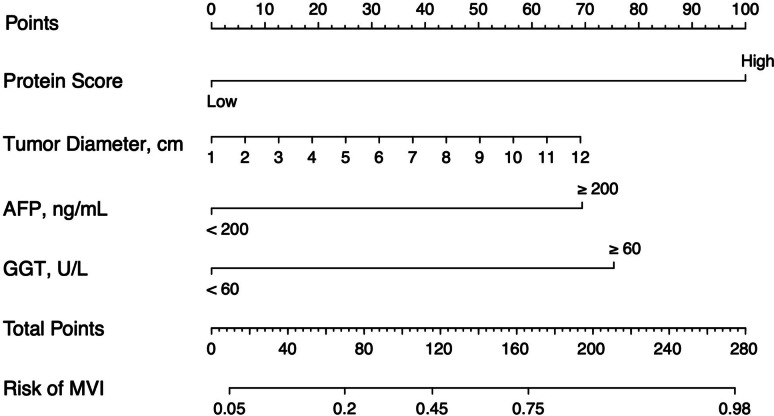
Nomogram to estimate the risk of MVI presence preoperatively in HCC patients

**Fig. 5:**
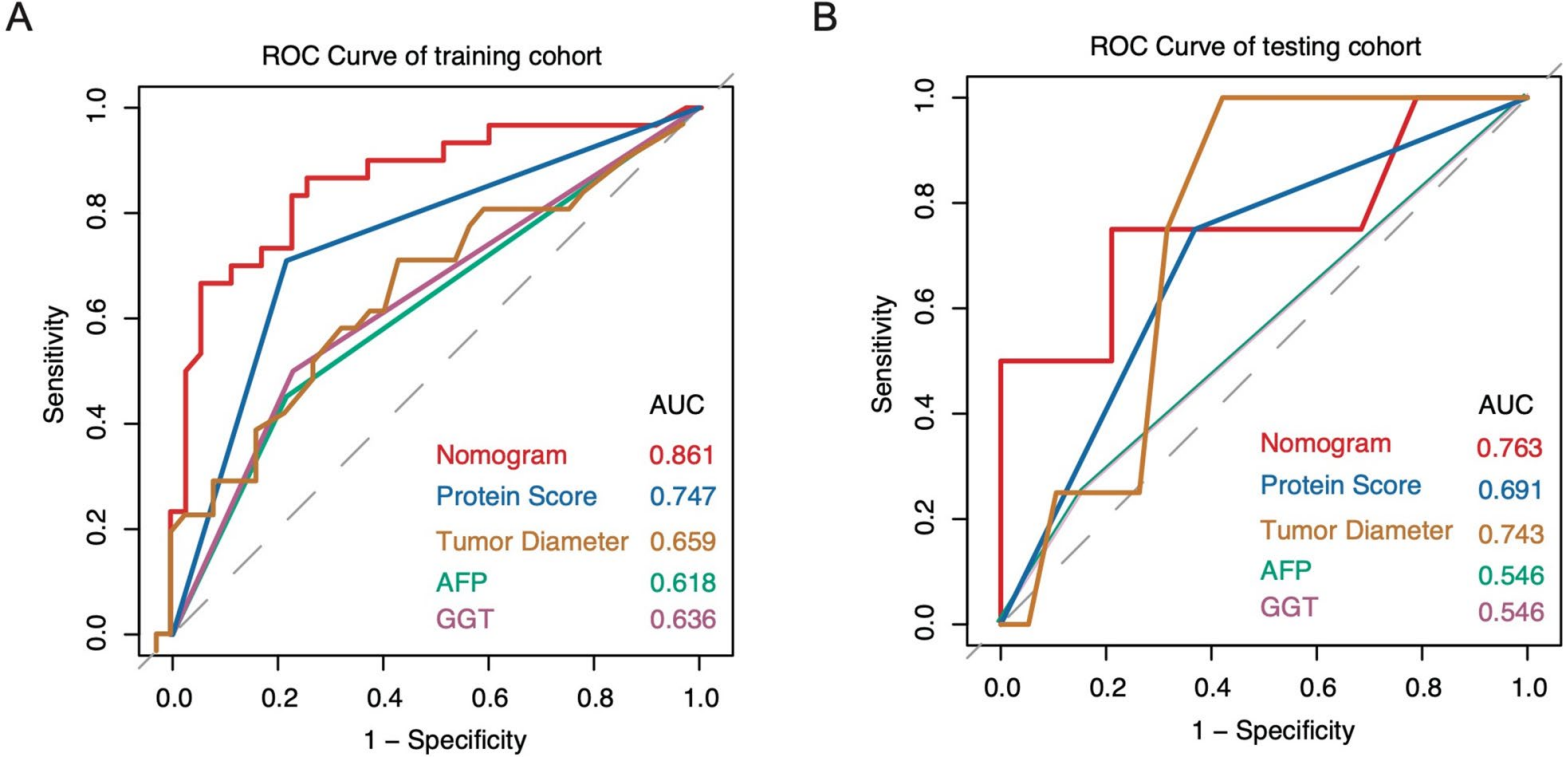
ROC Curves of nomograms for predicting MVI risk. **A** Training cohort. **B** Testing cohort

To provide a readable and quantitative measurement pre-operation to predict HCC patients’ risk of MVI, we constructed a comprehensive nomogram that integrated the four-protein-based protein score and preoperative clinical parameters. Univariate logistic analysis was performed to select the variables included in the nomogram. Variables with *p* < 0.05 were selected to enter the multivariate regression; according to the analysis results shown in Table 1, tumor diameter, serum AFP and GGT levels of patients, and protein score were independently associated with MVI. These independently associated risk factors were used to form the preoperative MVI prediction nomogram (Fig. 4). The predictive accuracy of the nomogram is shown in Fig. 5. The nomogram AUC was 0.809 in the training cohort, and its prediction performance was significantly higher than the protein score, combined clinical information, and single clinical parameter. The AUC in the testing cohort was 0.783. The nomogram shows good performance in predicting MVI in both the training cohort and the testing cohort, and it could predict the MVI status through the patients’ urinary proteomics before surgery.

**Table 1:** Logistic regression of MVI presence based on preoperative data in the training cohort

Variable	Univariate logistic OR (95% CI)	Regression *P*	Multivariate logistic OR (95% CI)	Regression *P*
**Sex**, male vs female	0.55 (0.13–2.42)	0.43		
**Age**, year	0.99 (0.94–1.03)	0.55		
**Tumor number**	0.82 (0.23–2.91)	0.76		
**Tumor diameter**, cm	1.26 (1.03–1.55)	0.02	1.197 (0.924–1.552)	0.17
**Capsule invasion**, Complete vs incomplete	2.27 (0.86–6.03)	0.1		
**WBCs**, 10^9^/L				
< 4 vs 4–10	0.83 (0.09–7.68)	0.87		
4–10 vs > 10	0.49 (0.08–3.21)	0.46		
**HGB**, g/L				
< 120 vs 120–160	1 (0.07–14.64)	1		
120–160 vs > 160	1.93 (0.52–7.18)	0.33		
**NEUT**, 10^9^/L				
< 2.04 vs 2.04–7.50	1 (0.12–8.31)	1		
2.04–7.50 vs > 7.50	0.8 (0.15–4.33)	0.8		
**MONO**, 0.12–0.8 vs > 0.8 × 10^9^/L	0.83 (0.11–6.25)	0.86		
**LYMPH**, < 0.80 vs 0.80–0.40 × 10^9^/L	2.65 (0.26–26.82)	0.41		
**PLT**, 10^9^/L				
< 100 vs 100–300	18781632.9 (0–Inf)	0.99		
100–300 vs > 300	13042800.6 (0–Inf)	0.99		
**PT(s)**, 10.0–13.2 vs > 13.2 S	1.15 (0.35–3.76)	0.82		
**APTT**, S				
< 20.9 vs 20.9–34.6	1.33 (0.07–26.62)	0.85		
20.9–34.6 vs > 34.6	1.75 (0.15–20.35)	0.65		
**D-D**, ≤ 0.55 vs > 0.55 mg/L FEU	0.42 (0.14–1.28)	0.13		
**GLU**, mmol/L				
< 3.89 vs 3.89–6.38	0 (0–Inf)	0.99		
3.89–6.38 vs > 6.38	1.26 (0.43–3.7)	0.67		
**CRE**, µmol/L				
< 57.0 vs 57.0–97.0	1.67 (0.19–14.27)	0.64		
57.0–97.0 vs > 97.0	1.44 (0.31–6.62)	0.64		
**ALB**, < 40.0 vs 40.0–55.0 g/L	0.79 (0.27–2.31)	0.66		
**ALT**, U/L				
< 9.0 vs 9.0–50.0	5,182,031.57 (0–Inf)	0.99		
9.0–50.0 vs > 50.0	0.64 (0.22–1.87)	0.42		
**AST**, U/L				
< 15.0 vs 15.0–40.0	0.9 (0.05–16.6)	0.94		
15.0–40.0 vs > 40.0	0.67 (0.23–1.94)	0.46		
**GGT**, 10.0–60.0 vs > 60.0	3.75 (0.29–47.99)	0.03	2.643 (0.04–179.11)	0.03
**TBIL**, 2–21 vs > 21 µmol/L	1.05 (0.26–4.32)	0.94		
**DBIL**, ≤ 5.1 vs > 5.1	0.88 (0.32–2.42)	0.8		
**ALP**, U/L				
< 45.0 vs 45.0–125.0	4 (0.21–75.66)	0.36		
45.0–125.0 vs > 125.0	1.69 (0.29–9.94)	0.56		
**CRP**, ≤ 0.6 vs > 0.6 mg/dl	1.08 (0.26–4.46)	0.91		
**AFP**, ≤ 200 vs > 200 ng/ml	2.99(1.04–8.57)	0.04	6.268 (1.40–28.09)	0.02
**CA199**, ≤ 27 vs > 27 U/ml	0.58 (0.2–1.67)	0.32		
**CEA**, ≤ 5.0 vs > 5.0 ng/ml	0.65 (0.18–2.38)	0.52		
**HBsAg**, positive vs negative	1.76 (0.57–5.49)	0.33		
**HBsAb**, Positive vs negative	1.25 (0.39–4.06)	0.71		
**HBeAg**, Positive vs negative	0.87 (0.27–2.85)	0.82		
**HBcAb**, Positive vs negative	1.57 (0.41–5.98)	0.5		
**Anti-HCV**, Positive vs negative	1.21 (0.23–6.49)	0.82		
**Protein score**, High vs low	2.72 (1.69–4.38)	0.0001	14.39 (3.47–59.65)	0.0001

### Immunoassay verification of urinary protein signatures.

To validate urinary signatures to distinguish the MVI status of HCC patients on a large scale, we developed a quantitative dot blot detection system using urine according to previous methods used with serum. The urinary protein amount was quantified by standard curves of each protein and then calibrated by the corresponding urine creatinine measurement.

**Fig. 6:**
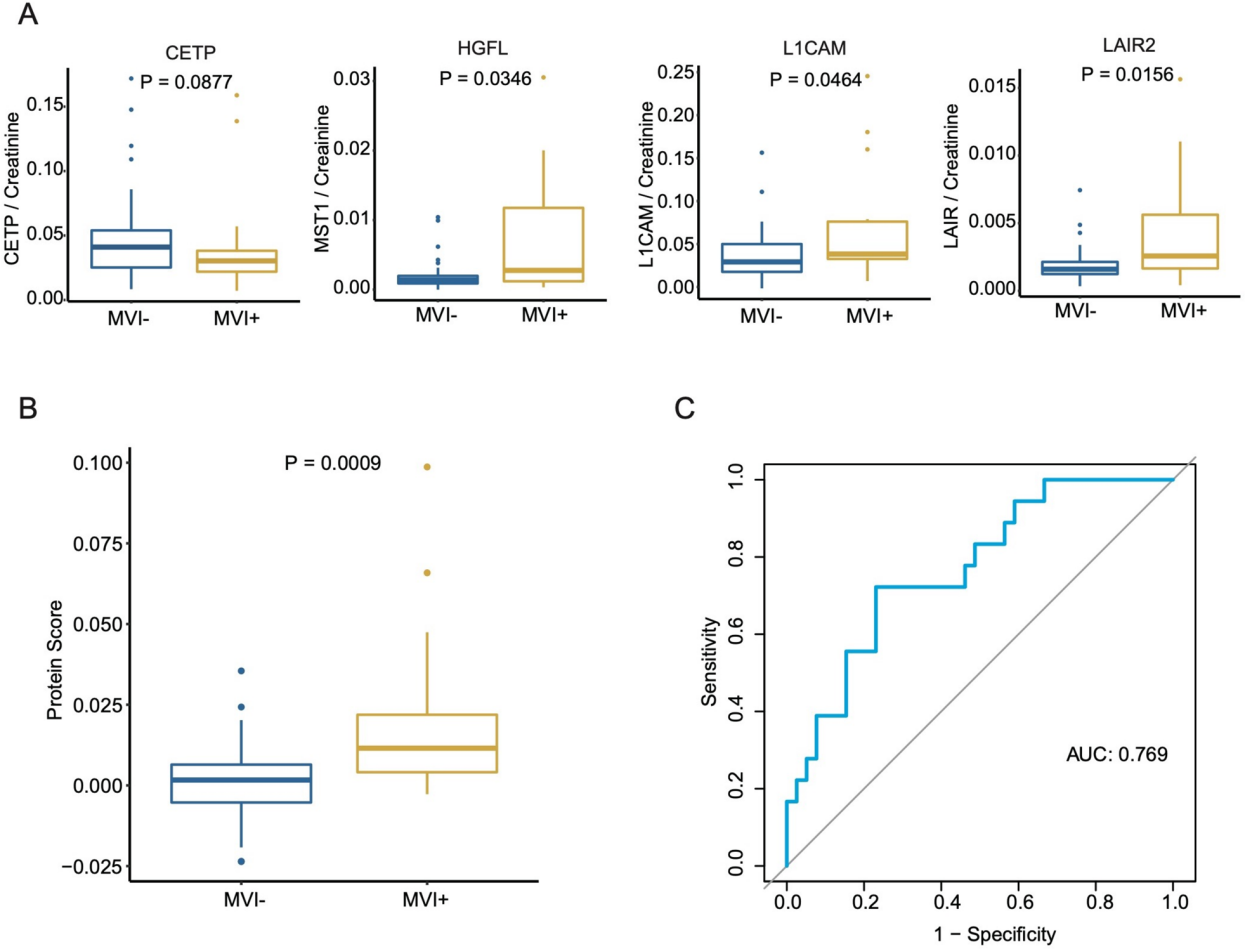
Independent urine verification of the urinary protein signature using ELISA. **A** The expression level of the four proteins in an independent HCC validation cohort. **B** Protein scores calculated from the protein expression level obtained by ELISA in the independent HCC validation cohort. **C** The ROC curve of protein scores in independent HCC validation cohort

A total of 57 urine samples of HCC patients were recruited in the immunoassay verification stage. The concentrations of *HGFL*, *L1CAM*, and *LAIR2* were significantly higher in the urine of HCC patients with MVI, and the concentrations of *CETP* were lower in the urine of HCC patients with MVI (Fig. 6A). The protein scores established by ELISA were also significantly higher in the MVI-positive group than in the MVI-negative group (Fig. 6B). In the testing cohort, the protein scores displayed a C index of 0.769 for the estimation of MVI risk (Fig. 6C).

Based on these preoperative predictions, the nomogram might serve as a tool to select patients for randomized clinical trials for evaluating the efficacy of liver resection in patients with HCC and different risks of MVI.

## Discussion

The presence of MVI significantly worsens the surgical outcomes of HCC, which was a risk factor of tumor recurrence and overall survival [18]. Predicting the MVI status of HCC patients pre-surgery could help clinicians choose precise surgical methods for individualized treatment. Previous studies have attempted to use preoperative imaging or serum biomarkers to predict MVI before surgery [19], but there are still problems such as low predictive efficiency and large-scale verification. One study reported a model that incorporated factors (number of tumors, diameter, and serum α-fetoprotein level) in the preoperative risk estimation of MVI [20]. However, the use of this model requires specific computer software, thus limiting its wide use. One study reported a nomogram for predicting MVI status in hepatitis B virus-related patients based on clinical features, but it was only based on clinical data and needs to be supplemented with specific markers to improve accuracy [21].

Human body fluids are considered rich sources of potential disease biomarkers, they have potential applicability for the early detection of disease and monitoring of progression. Urine with less complexity than serum is a promising study medium for the discovery of novel biomarkers in many human diseases, such as prostate cancer and bladder cancer [22, 23]. With the increasing progress of mass spectrometry (MS) and proteomics technology, proteomics provides a reliable way for the discovery of body fluid biomarkers.

In this study, we systematically analyzed urinary proteins for the HCC preoperative MVI prediction model using a combination of quantitative proteomics and immunoassays. To date, this is the largest and most comprehensive study to identify non-invasive biomarkers for HCC.

In the MVI risk estimation nomogram, large tumor size and high serum levels of AFP and GGT concentrations have been reported to increase the possibility of MVI in HCCs [24, 25]. In addition, we demonstrated that combined urine protein signatures are more beneficial for the preoperative prediction of patients’ MVI status. *CETP* is involved in lipid transport [26], and studies have reported that MVI-positive patients have a more active metabolic state, which deserves further attention. As a hepatocyte growth factor-like protein, *HGFL*’s important role in the proliferation and metastasis of HCC has been reported in many literatures [27]. As a neuro-adhesion-related molecule, the function of *L1CAM* in tumor metastasis has been reported [28, 29], and the MVI status of liver cancer patients is closely related to recurrence and metastasis. As a secreted protein, *LAIR2* can lead to tumor immune escape [30]. Our previous studies have reported that there are different immune landscapes in HCC patients with different MVI statuses.

For clinical use of the model, we summarized the predictive value in estimating the risk of MVI. Based on these preoperative predictions, the nomogram might serve as a tool to select patients for randomized clinical trials for evaluating the efficacy of liver resection in patients with early HCC and different risks of MVI. In addition, the suitability of liver transplantation can be assessed because the absence of MVI is an essential variable in the new criteria for this treatment. The preoperatively estimated MVI risk status can be used in recruiting patients into studies on neoadjuvant therapy for HCC.

In this study, we used urinary proteomics technology to analyze the difference in urinary protein expression between HCC patients and healthy controls, the nomogram combined the urinary proteins and clinicopathologic factors showed a good predictive performance for the accurate detection of MVI pre-surgery in HCC patients. Our results showed that this nomogram can successfully categorize patients into high-risk MVI and low-risk MVI with large differences.

Our study has some limitations. First, the mechanism of action of the four proteins in the occurrence of MVI needs to be further explored. Second, this analysis is based on data from a single institution; it will be necessary to validate results from other centers. Finally, prospective studies are needed to further confirm the reliability of the nomogram.

Our study had some limitations. First, this analysis was based on data from a single institution, it is necessary to validate the results from other centers. Second, collecting consecutive urine samples may be of great help to our work and increase the confidence of our model. Finally, a panel based on biomarkers is required to design to further confirm the reliability of the nomogram.

### Supplementary Information

**Additional file 1: Figure S1.** Pathological and prognostic characteristics of HCC patients with different MVI status. A, H&E staining images of representative HCC patients with different MVI states. MVIs has been marked in the figure with arrows. B, C, Kaplan-Meier plots of OS/DFS times for patients with HCC stratified by MVI. **Figure S2.** An Overview of urinary proteome identified proteins. A, Protein expression levels identified by urinary proteomics. B, The number of proteins identified in three sets of samples. C, Venn diagram summary of the number of proteins identified by urinary proteomics. **Figure S3.** Proteomics Features of three groups. A, Principal component analysis (PCA) of urinary proteomic data. B, WGCNA performs soft threshold screening. C, Protein expression levels of representative modules in three groups. D, Volcano plot of differential expressed proteins between MVI and non-MVI group. **Figure S4.** Expression levels and prognostic value of characteristic proteins. A, Correlation of characteristic protein expression. B, Forest plot of multivariate logistic regression of signature proteins. C, Prognostic value of expression levels of individual signature proteins.

**Additional file 2.** Patient MVI status characteristics.

**Additional file 3.** Differential expressed proteins between the MVI, non-MVI, and healthy sub-cohorts.

## Data Availability

The MS proteomics data generated in this study have been deposited to the ProteomeXchange Consortium (http://proteomecentral.proteomexchange.org) via the iProX partner repository (http://iprox.cn) with the dataset identifier PXD037409 and IPX0005188000.

## Abbreviations

DFS: Disease-free survival
ELISA: Enzyme-linked immunosorbent assay
HCC: Hepatocellular carcinoma
MS: Mass spectrometry
MVI: Microvascular invasion
OS: Overall survival
PLS-DA: Partial least squares discriminant analysis
ROC: Receiver operating characteristic
WGCNA: Weight gene co-expression network analysis
