# Supplementary material for: Noninvasive urinary protein signatures combined clinical information associated with microvascular invasion risk in HCC patients
Source: BMC Med. 2023 Dec 5;21:481. doi: 10.1186/s12916-023-03137-6 (PMC10696877; doi:10.1186/s12916-023-03137-6)
Supplement: Supplementary file 1 — Additional file 1: Figure S1. Pathological and prognostic characteristics of HCC patients with different MVI status. A, H&E staining images of representative HCC patients with different MVI states. MVIs has been marked in the figure with arrows. B, C, Kaplan-Meier plots of OS/DFS times for patients with HCC stratified by MVI. Figure S2. An Overview of urinary proteome identified proteins. A, Protein expression levels identified by urinary proteomics. B, The number of proteins identified in three sets of samples. C, Venn diagram summary of the number of proteins identified by urinary proteomics. Figure S3. Proteomics Features of three groups. A, Principal component analysis (PCA) of urinary proteomic data. B, WGCNA performs soft threshold screening. C, Protein expression levels of representative modules in three groups. D, Volcano plot of differential expressed proteins between MVI and non-MVI group. Figure S4. Expression levels and prognostic value of characteristic proteins. A, Correlation of characteristic protein expression. B, Forest plot of multivariate logistic regression of signature proteins. C, Prognostic value of expression levels of individual signature proteins. [file 12916_2023_3137_MOESM1_ESM.pdf]

Additional file 1 for

Noninvasive urinary protein signatures combined clinical information associated with Microvascular Invasion Risk in HCC patients

Yaru Wang[1,2,6,#], Bo Meng[3,5,#], Xijun Wang[2], Anke Wu[1], Xiaoyu Li[3,4], Xiaohong Qian[3], Jianxiong Wu[1], Wantao Ying[3,4,*], Ting Xiao[2,*], Weiqi Rong[1,*]

Correspondence to: dr_rongweiqi@163.com, xiaot@cicams.ac.cn, yingwantao@nspsb.org.cn

This PDF file includes:

Figures S1 to S4

Table S1

Figure S1

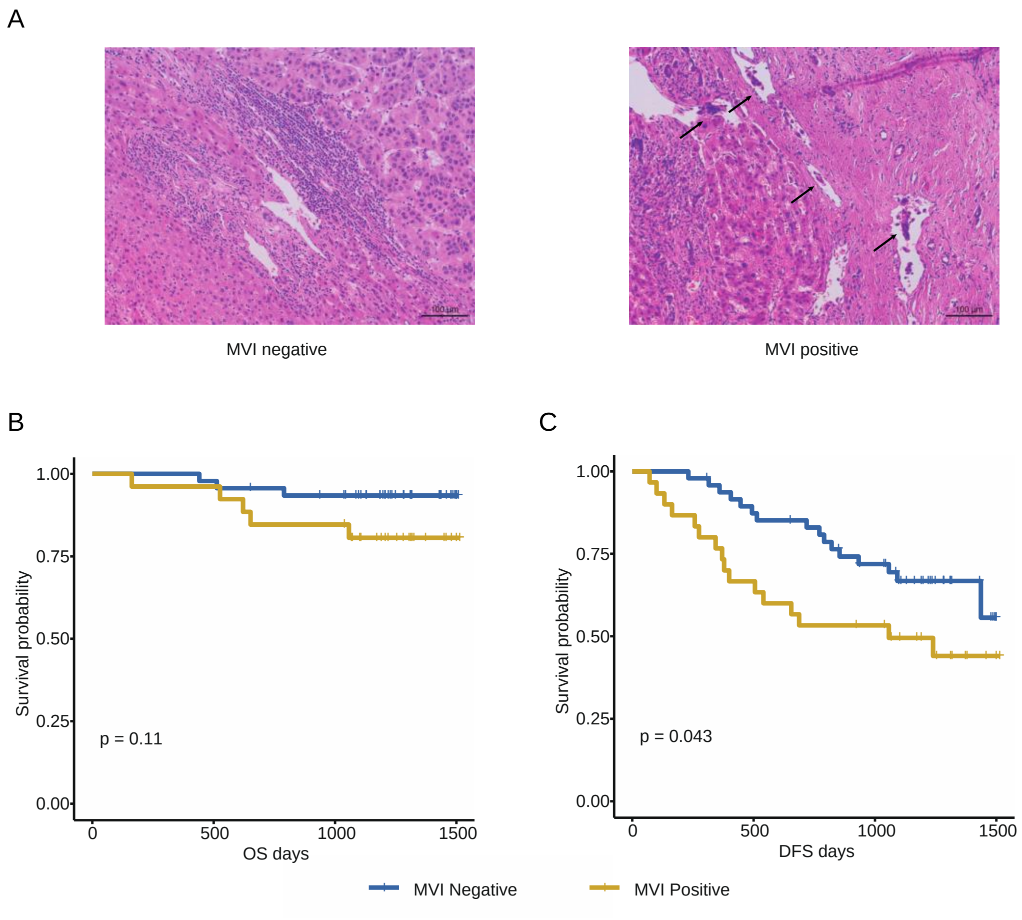

Figure S1. Pathological and prognostic characteristics of HCC patients with different MVI status. A, H&E staining images of representative HCC patients with different MVI states. MVIs has been marked in the figure with arrows. B, C, Kaplan-Meier plots of OS/DFS times for patients with HCC stratified by MVI.

Figure S2

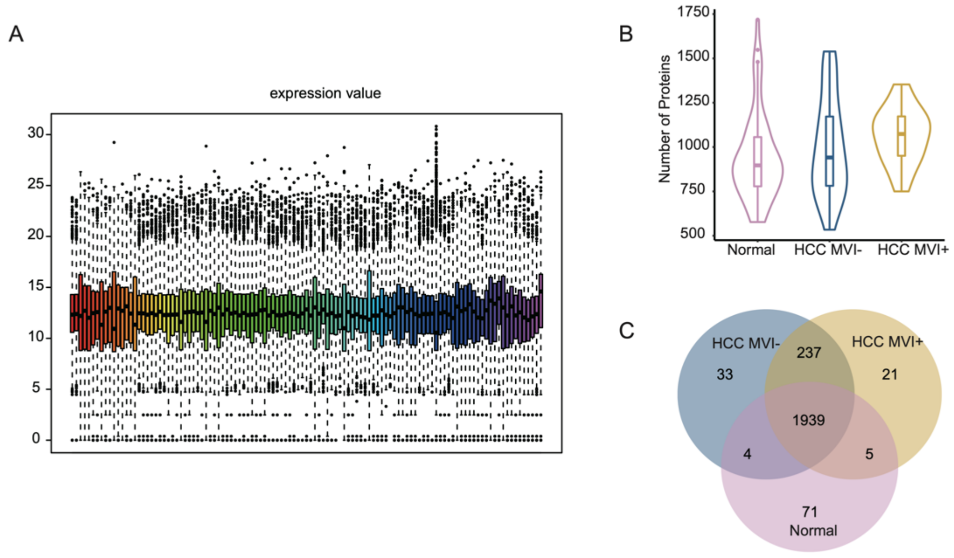

Figure S2. An Overview of urinary proteome identified proteins. A, Protein expression levels identified by urinary proteomics. B, The number of proteins identified in three sets of samples. C, Venn diagram summary of the number of proteins identified by urinary proteomics.

Figure S3

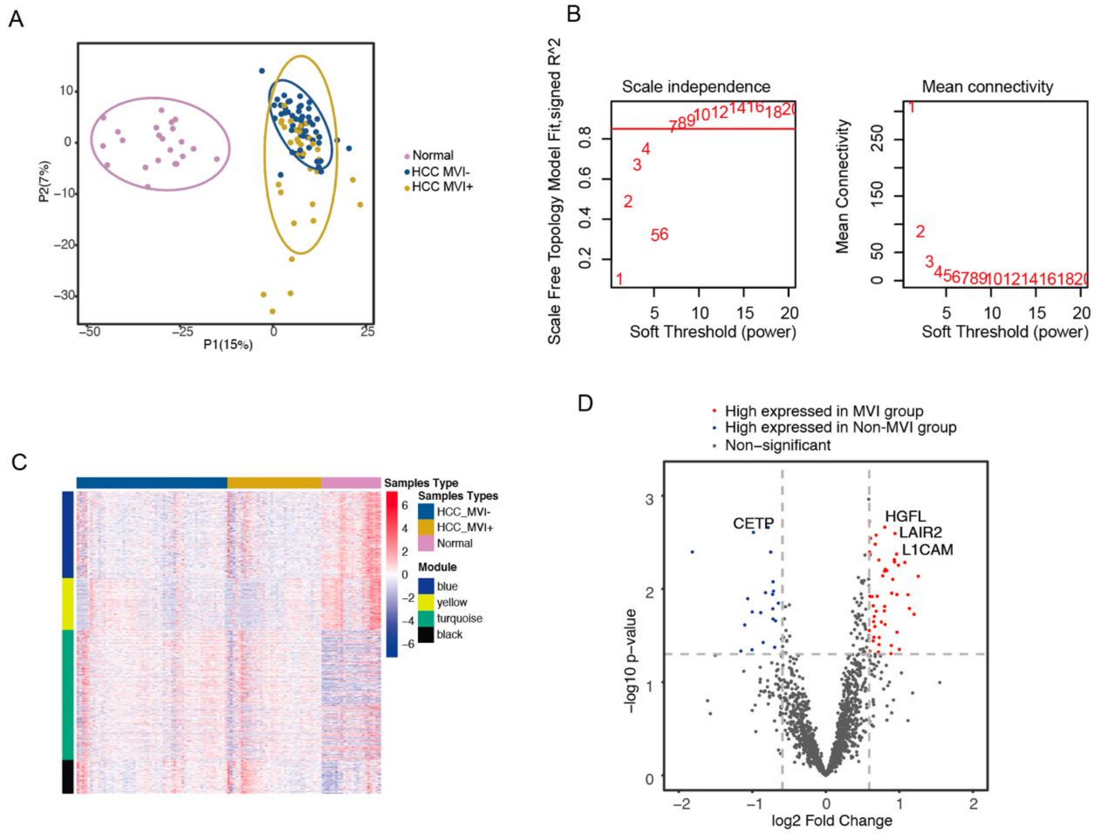

Figure S3. Proteomics Features of three groups. A, Principal component analysis (PCA) of urinary proteomic data. B, WGCNA performs soft threshold screening. C, Protein expression levels of representative modules in three groups. D, Volcano plot of differential expressed proteins between MVI and non-MVI group.

Figure S4

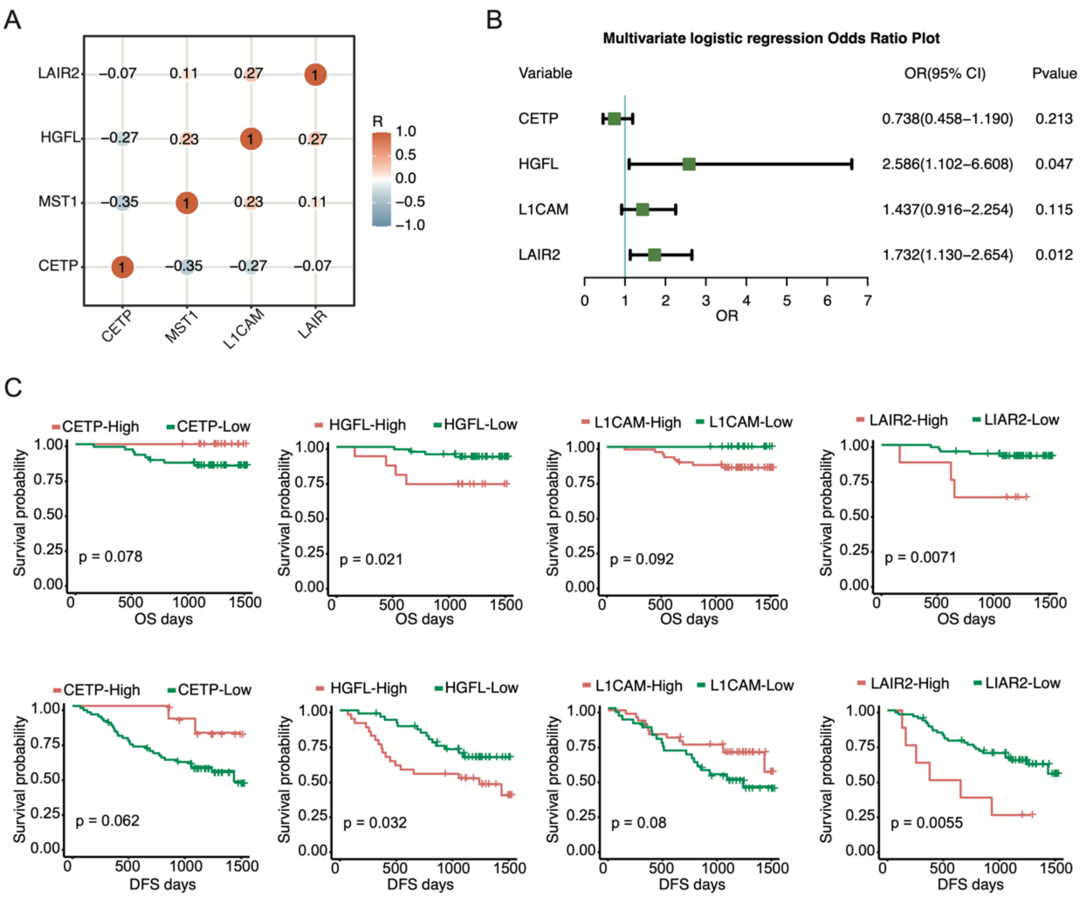

Figure S4, Expression levels and prognostic value of characteristic proteins. A, Correlation of characteristic protein expression. B, Forest plot of multivariate logistic regression of signature proteins. C, Prognostic value of expression levels of individual signature proteins.

Table S1. Characteristics of HCC patients analyzed by proteomics

	Cohort, No. (%)		
Variable	Training (n = 68)	Testing (n = 23)	P Value
**Sex**			0.731
Male	59 (86.8)	19 (82.6)	
Female	9 (13.2)	4 (17.4)	
**Age, mean (SD), y**	59.43 (9.89)	61.26 (11.77)	0.466
**Tumor number**			
Solitary	56(82.4)	22(95.7)	0.172
Multiple	12(17.6)	1(4.3)	
**Tumor diameter, mean (SD), cm**	4.51 (2.65)	4.62 (1.99)	0.858
**Tumor capsule**			0.676
Complete	36 (52.9)	14 (60.9)	
Incomplete	32 (47.1)	9 (39.1)	
**WBCs, 10^9/L**			0.356
>10	5 (7.4)	4 (17.4)	
4-10	54 (79.4)	17 (73.9)	
<4	9 (13.2)	2 (8.7)	
**HGB, g/L**			0.893
>160	12 (17.6)	3 (13.0)	
120-160	53 (77.9)	19 (82.6)	
<120	3 (4.4)	1 (4.3)	
**NEUT, 10^9/L**			0.46
>7.50	6 (8.8)	4 (17.4)	
2.04-7.50	54 (79.4)	16 (69.6)	
<2.04	8 (11.8)	3 (13.0)	
**MONO, 10^9/L**			1
>0.80	4 (5.9)	1 (4.3)	
0.12-0.80	64 (94.1)	22 (95.7)	
**LYMPH, 10^9/L**			0.108
0.80-4.00	64 (94.1)	19 (82.6)	
<0.80	4 (5.9)	4 (17.4)	
**PLT, 10^9/L**			0.266
>300.0	2 (2.9)	0 (0.0)	
100.0-300.0	55 (80.9)	22 (95.7)	
<100.0	11 (16.2)	1 (4.3)	
**PT, S**			1
>13.2	14 (20.6)	4 (17.4)	
10.0-13.2	54 (79.4)	19 (82.6)	

**APTT, S**			0.368
>34.6	3 (4.4)	2 (8.7)	
20.9-34.6	60 (88.2)	21 (91.3)	
<20.9	5 (7.4)	0 (0.0)	
**D-D, mg/L FEU**			1
>0.55	18 (26.5)	6 (26.1)	
≤0.55	50 (73.5)	17 (73.9)	
**GLU, mmol/L**			0.405
>6.38	19 (27.9)	10 (43.5)	
3.89-6.38	48 (70.6)	13 (56.5)	
<3.89	1 (1.5)	0 (0.0)	
**CRE, umol/L**			0.536
>97	8 (11.8)	4 (17.4)	
57-97	54 (79.4)	16 (69.6)	
<57	6 (8.8)	3 (13.0)	
**ALB, g/L**			0.785
40.0-55.0	50 (73.5)	18 (78.3)	
<40.0	18 (26.5)	5 (21.7)	
**ALT, U/L**			0.524
>50.0	19 (27.9)	7 (30.4)	
9.0-50.0	48 (70.6)	15 (65.2)	
<9.0	1 (1.5)	1 (4.3)	
**AST, U/L**			1
>40.0	19 (27.9)	6 (26.1)	
15.0-40.0	47 (69.1)	17 (73.9)	
<15.0	2 (2.9)	0 (0.0)	
**GGT, U/L**			0.124
>60.0	23 (35.4)	4 (17.4)	
10.0-60.0	42 (64.6)	19 (82.6)	
**TBIL, umol/L**			0.442
>21	9 (13.2)	1 (4.3)	
2-21	59 (86.8)	22 (95.7)	
**DBIL, umol/L**			0.319
>5.1	45 (66.2)	12 (52.2)	
≤5.1	23 (33.8)	11 (47.8)	
**ALP, U/L**			0.859
>125.0	6 (8.8)	2 (8.7)	
45.0-125.0	59 (86.8)	21 (91.3)	
<45.0	3 (4.4)	0 (0.0)	
**CRP, mg/dl**			1
>0.6	9 (13.8)	3 (13.0)	
≤0.6	56 (86.2)	20 (87.0)	
**AFP, ng/mL**			0.47

>7.0	37 (54.4)	10 (43.5)	
≤7.0	31 (45.6)	13 (56.5)	
**CA199, U/mL**			0.052
>27.0	20 (29.4)	2 (8.7)	
≤27.0	48 (70.6)	21 (91.3)	
**CEA, ng/mL**			1
>5.0	11 (16.2)	3 (13.0)	
≤5.0	57 (83.8)	20 (87.0)	
**GGT, U/L**			0.124
>60.0	23 (35.4)	4 (17.4)	
10.0-60.0	42 (64.6)	19 (82.6)	
**HBsAg**			0.18
negative	14 (20.6)	9 (39.1)	
positive	51 (75.0)	14 (60.9)	
**HBsAb**			0.709
negative	51 (75.0)	19 (82.6)	
positive	14 (20.6)	4 (17.4)	
**HBeAg**			0.531
negative	51 (75.0)	20 (87.0)	
positive	14 (20.6)	3 (13.0)	
**HCV.Ab**			0.687
negative	62 (91.2)	20 (87.0)	
positive	6 (8.8)	3 (13.0)	
